# Trends in cancer-related suicide in the United States: a population-based epidemiology study spanning 40 years of data

**DOI:** 10.1038/s41398-024-02917-9

**Published:** 2024-05-27

**Authors:** Qiang Liu, Zheng Qu, Hao Dong, Yihang Qi, Juan Wu, Wenxiang Zhang, Xiangyu Wang, Zhongzhao Wang, Yi Fang, Jing Wang

**Affiliations:** 1 Department of Breast Surgical Oncology, National Cancer Center/National Clinical Research Center for Cancer/Cancer Hospital, Chinese Academy of Medical Sciences and Peking Union Medical College, Beijing, 100021 China; 2 Department of Breast Surgery, Peking Union Medical College Hospital, Beijing, China

**Keywords:** Psychiatric disorders, Scientific community

## Abstract

Large cohort studies examining trends in cancer-related suicide are lacking. We analyzed data from the Surveillance, Epidemiology, and End Results (SEER) database, encompassing a total of 4,870,410 patients diagnosed with cancer from 1975 to 2017 in the United States. Joinpoint regression was used to estimate the annual percent change (APC) and average annual percentage change (AAPC) of age-adjusted rates of suicide. In the past 40 years, we revealed a gradual increase in cancer-related suicide rates from 1975 to 1989, followed by a gradual decrease from 1989 to 2013, and a marked decrease from 2013 to 2017. These trends suggested the potential impact of advancements in psychosocial care for patients with cancer in contributing to the observed decrease in suicide rates.

## Introduction

Cancer, the second leading cause of death across all age groups in the United States (US) [1], is often accompanied by a higher risk of suicide. Suicide was the tenth leading cause of death among individuals of all ages, with 47,511 suicide cases recorded in 2019 [1]. Furthermore, suicide is recognized as a significant contributor to premature mortality [2–4]. Patients with cancer face an elevated risk of suicide, primarily attributable to psychological distress, advert effects from multiple treatments, diminished quality of life, and uncontrolled and intense cancer-related pain [5–8]. The shift toward a bio-psycho-social medical model has highlighted the importance of assessing and managing the risk of suicide and implementing suicide prevention strategies and psychological interventions for cancer patients [4, 9–11].

With advancements in cancer screening and treatments and increased awareness regarding the importance of providing psychological support to patients with cancer, the trends of cancer-related suicide among patients with cancer in the US have changed. However, current evidence regarding these trends is scarce. Only two previous studies have attempted to investigate this issue. One study conducted by Han et al. using the Multiple Cause of Death database reported a decreasing trend in suicides among individuals with cancer from 1999 to 2018. While this study was interesting, it did not specifically reflect the trends among patients with cancer; hence, its findings may not be applicable [12]. Another study by Violette et al. analyzed data from the Surveillance, Epidemiology, and End Results (SEER) database, focusing on 467,368 women diagnosed with uterine cancer, ovarian cancer, or cervical cancer from 1973 to 2013. They reported a downward trend in suicide rates in this specific subset of gynecologic malignancies [13]. However, the broader profile of suicide trends among patients with cancer in the US over the past decades remains unknown.

Based on the current research status, we propose the hypothesis that trends of cancer-related suicide in the US exhibit corresponding changes in line with shifts in cancer treatment and prevention strategies. Analyzing the suicide trends within the cancer population can provide valuable insights into potential suicide risks, identify changes in trends, and guide clinical practices within the context of the social psychological medical model. Furthermore, such analyses can promote the improvement of relevant policies aimed at improving the well-being and care of patients with cancer.

## Methods

### Data source and study population

We examined the age-adjusted rates and trends of cancer-related suicide in the US over a 40-year period, specifically from 1975 to 2017. All data were accessed from the SEER Research Plus Data [14], which accounts for approximately 10% of the US population (based on the 2010 census). The SEER database is a publicly available, federally funded cancer reporting system that represents a collaboration between the US Centers for Disease Control and Prevention, the National Cancer Institute, and regional and state cancer registries. In contrast to other commonly used datasets, the SEER database is considered population-based, as it collects information on all cancer cases within specific regions and/or defined racial/ethnic population. Therefore, it serves as a nationally representative, population-based cancer reporting system that includes all cancer cases within specific geographic regions of the US [15, 16].

Age-adjusted suicide rates for the total US population were analyzed using the US mortality data, which is collected and maintained by the National Center for Health Statistics. The inclusion criteria for the study were defined as follows: only patients diagnosed with cancer, those with cancer originating from any site, and those diagnosed from 1975 to 2017. Finally, a total of 4,870,410 patients were identified, among whom 8114 died by suicide. Cases were identified as suicide-related deaths only if the “COD to site record” class in the SEER program was coded as “Cause of Death (COD) and Follow-up. COD to site recode” = “Suicide and Self-Inflicted Injury’”. The requirement of obtaining written informed consent from the participants was waived as the study used a deidentified publicly available registry database.

### Statistical analysis

To assess the trend of cancer-related suicide within the general US population, we used a standardized method using the SEER*stat software [17]. Age-adjusted rates were calculated and standardized to the US standard population in the year 2000 and expressed per 100,000 person-years, as this approach is widely accepted and statistically robust practices. Annual percent change (APC) and average annual percentage change (AAPC) were calculated using the standardized method implemented in the Joinpoint Trend Analysis Software [18]. APC measures the change in rates over time, assuming a constant percent change relative to the rate of the previous year. AAPC, on the other hand, is a summary measure of the average APCs over a pre-specified fixed interval, allowing for a single number to represent the trend over multiple years. In our analyses, we selected the best-fitting log-linear regression model as the final model to identify joinpoints where APCs changed significantly, and the maximum joinpoints were set as 2. Student’s *t*-tests were performed to determine whether the APCs were statistically significant. All statistical analyses were performed using the Surveillance Research Program, National Cancer Institute SEER*Stat software (seer.cancer.gov/seerstat) version 8.3.9, the Joinpoint Regression Program, Version 4.8.0.1 - April 2020) developed by the Statistical Methodology and Applications Branch, Surveillance Research Program, National Cancer Institute, and R Statistical Software version 4.0.3 (R Foundation for Statistical Computing). All statistical tests were two-sided, and *P* < .05 was considered as the cutoff value for determining statistical significance.

## Results

### Overall findings and baseline characteristics

**Fig. 1:**
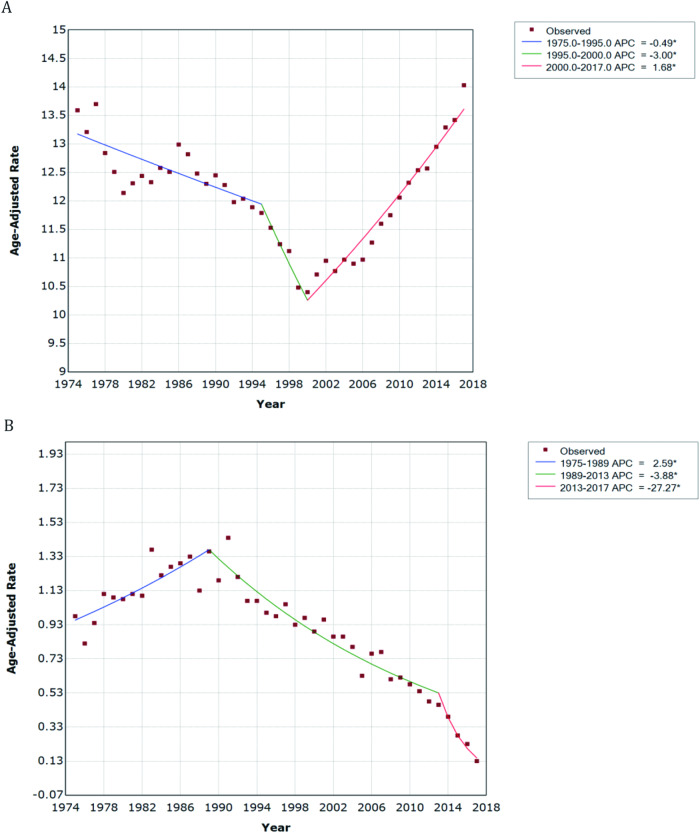
Age-adjusted suicide rate per 100,000 person-years and suicide trend in the past 40 years. **A** Suicide rates and trends among the total US population (**B**) Cancer-related suicide rates and trends in the US. APC Annual percentage change. *Indicates that the APC is significantly different from zero at the alpha = 0.05 level.

Among the 4,870,410 patients (51.2% female and 48.8% male) diagnosed with cancer from 1975 to 2017, a total of 8114 patients died by suicide. The majority of these were male (81.7%), Caucasian (92.8%), and aged between 50 and 79 years (72.5%). The age-adjusted rates of cancer-related suicide categorized by the characteristics of the study population are shown in Table 1. Overall, we found that the trends in suicide rates varied considerably between patients with cancer and the general US population (Fig. 1). The results of joinpoint regression analysis are shown in Table 2. We found a significant increasing trend of suicide rates in the US population as a whole since the year 2000 (APC, 1.7%; 95%CI, 1.5% to 1.9%; *P* < 0.001). Notably, there has been a decreasing trend in cancer-related suicide rates since 1989, especially in the most recent five years (AAPC, −27.3%; 95%CI, −37.2% to −15.8%, *P* < 0.05). We also found variations in the trends of cancer-related suicide rates based on sex, race, age, and registry center. In the most recent five years (2013–2017), significant declines in cancer-related suicide rates were observed among male patients (AAPC, −20.4%; 95%CI, −27.0% to −13.1%; *P* < 0.05), Caucasian patients (AAPC, −25.5%; 95%CI, −35.7% to −13.8%, *P* < 0.05), and patients aged 15 to 49 years (AAPC, −16.8%; 95%CI, −26.3% to −6.2%; *P* < 0.05) and 60 to 79 years (AAPC, −34.0%; 95%CI, − 48.5% to −15.4%; *P* < 0.05 for 60–69 years and 95CI% −29.0% to 7.2% for 70–79 years). Similar declining trends were observed in specific registry centers, including San Francisco-Oakland SMSA, Connecticut, Utah, and Atlanta (Metropolitan) (Table 2).

**Table 1:** Demographic characteristics and age-adjusted cancer-related suicide rates per SEER Data, 1975–2017.

Characteristics	Patients with cancer	Suicides, No. (%)	Age-adjusted rate, per 100,000 (95% CI)
**Overall**	4870410 (100)	8114 (100)	0.76 (0.74–0.78)
**Sex**			
Male	2492120 (51.2)	6633 (81.7)	1.4 (1.36–1.43)
Female	2378290 (48.8)	1481 (18.3)	0.26 (0.25–0.28)
**Race**			
Caucasian	4065373 (83.5)	7517 (92.8)	0.86 (0.84–0.88)
African American	456176 (9.4)	253 (3.1)	0.26 (0.23–0.30)
Other	324454 (6.7)	327 (4.1)	0.33 (0.29–0.36)
**Age**			
15–49	690099 (14.2)	1438 (17.8)	0.27 (0.26–0.29)
50–59	846062 (17.4)	1479 (18.3)	1.18 (1.13–1.25)
60–69	1273999 (26.2)	2294 (28.4)	2.59 (2.48–2.70)
70–79	1239232 (25.4)	2079 (25.8)	3.69 (3.53–3.85)
> 80	786521 (16.1)	782 (9.7)	2.39 (2.23–2.57)
**Registry**			
San Francisco-Oakland SMSA	754866 (15.5)	1528 (18.8)	0.9 (0.86–0.95)
Connecticut	744029 (15.3)	743 (9.2)	0.48 (0.45–0.52)
Detroit (Metropolitan)	840258 (17.3)	1092 (13.5)	0.64 (0.61–0.68)
Hawaii	207104 (4.3)	312 (3.8)	0.62 (0.55–0.69)
Iowa	634916 (13.0)	959 (11.8)	0.7 (0.66–0.75)
New Mexico	273923 (5.6)	765 (9.4)	1.09 (1.01–1.17)
Seattle (Puget Sound)	746086 (15.3)	1511 (18.6)	0.97 (0.92–1.02)
Utah	268980 (5.5)	582 (7.2)	0.84 (0.77–0.91)
Atlanta (Metropolitan)	400248 (8.2)	622 (7.7)	0.69 (0.64–0.75)

**Table 2:** Trends in cancer-related suicide rates in the United States from 1975 to 2017.

	Trend 1			Trend 2			Trend 3			Fixed interval 2013–2017
	Years	APC,% (95% CI)	*P*	Years	APC,% (95% CI)	*P*	Years	APC,% (95% CI)	*P*	AAPC,% (95% CI)
Overall	1975–1989	2.6 (1.4,3.8)	<0.001	1989–2013	−3.9 ( − 4.4, − 3.4)	<0.001	2013–2017	−27.3 ( − 37.2, − 15.8)	<0.001	−27.3 (−37.2, − 15.8)
Sex										
Male	1975–1989	3.5 (2.3,4.8)	<0.001	1989–2012	−4.2 ( − 4.7, − 3.6)	<0.001	2012–2017	−20.4 ( − 27.0, − 13.1)	<0.001	−20.4 (−27.0, − 13.1)
Female	1975–2007	−2.0 ( − 2.8, −1.3)	<0.001	2007–2017	−9.8 ( − 15.4, − 3.8)	0.002				−9.8 (−15.4, − 3.8)
Race										
Caucasian	1975–1989	2.9 (1.6,4.2)	<0.001	1989–2013	−3.7 ( − 4.3, − 3.2)	<0.001	2013–2017	−25.2 ( − 35.7, − 13.8)	<0.001	−25.5 (−35.7, − 13.8)
African American	1975–1994	1.6 ( − 2.0,5.3)	0.373	1994–2017	−5.6 ( − 8.2, − 2.9)	<0.001				−5.6 (−8.2, − 2.9)
Other	1975–2000	0.0 ( − 1.9,1.9)	0.996	2000–2017	−7.6 ( − 10.4, − 4.7)	<0.001				−7.6 (−10.4, −4.7)
Age										
15–49	1975–1986	1.4 ( − 1.8,4.8)	0.385	1986–2010	−3.5 ( − 4.5, − 2.5)	<0.001	2010–2017	−16.8 ( − 26.3, − 6.2)	0.004	−16.8 (−26.3, − 6.2)
50–59	1975–2002	−0.5 ( − 1.4,0.4)	0.306	2002–2017	−7.3 ( − 9.8, − 4.8)	<0.001				−7.3 (−9.8, − 4.8)
60–69	1975–1991	2.4 (0.9,4.0)	0.002	1991–2013	−4.9 ( − 5.9, − 3.9)	<0.001	2013–2017	−34.0 ( − 48.5, − 15.4)	0.002	−34.0 (−48.5, − 15.4)
70–79	1975–1986	6.0 (2.2,9.9)	0.002	1986–2011	−3.3 ( − 4.3, − 2.4)	<0.001	2011–2017	−18.9 ( − 29, − 7.2)	0.003	−18.9 (−29.0, − 7.2)
>80	1975–1988	5.6 (2.1,9.1)	0.002	1988–2017	−3.5 ( − 4.3, − 2.7)	<0.001				−3.5 (−4.3, − 2.7)
Registry										
San Francisco-Oakland SMSA	1975–1989	1.7 ( − 0.4,4.1)	0.168	1989–2014	−5.2 ( − 6.4, − 4.0)	<0.001	2014–2017	−45.3 ( − 73.9,14.7)	0.107	−37.2 (−63.3,7.3)
Connecticut	1975–1979	25.2 ( − 0.2,57.8)	0.052	1979–2013	−2.5 ( − 3.3, − 1.6)	<0.001	2013–2017	−44.2 ( − 65.9, − 8.4)	0.022	−44.2 (−65.9, − 8.4)
Detroit (Metropolitan)	1975–1983	8.1 (1.6, 15.1)	0.016	1983–2007	−2.6 ( − 3.7, − 1.4)	<0.001	2007–2017	−10.5 ( − 16.1, − 4.5)	0.001	−10.5 (−16.1, − 4.5)
Hawaii	1975–1991	2.3 ( − 2.2,7.0)	0.310	1991–2017	−4.7 ( − 6.6, − 2.8)	<0.001				−4.7 (−6.6, − 2.8)
Iowa	1975–1980	7.8 ( − 3.2,20.0)	0.165	1980–2006	−1.2 ( − 2.0, − 0.3)	0.007	2006–2017	−8.6 ( − 12.3, − 4.8)	<0.001	−8.6 (−12.3, − 4.8)
New Mexico	1975–2003	1.5 (0.2,2.8)	0.030	2003–2017	−11.1 ( − 14.4, − 7.7)	<0.001				−11.1 (−14.4, − 7.7)
Seattle (Puget Sound)	1975–1987	4.7 (1.6,7.9)	0.004	1987–2011	−4.3 ( − 5.3, − 3.3)	<0.001	2011–2017	−13.7 ( − 22.9, − 3.5)	0.011	−13.7 (−22.9, − 3.5)
Utah	1975–2013	−1.6 ( − 2.4, − 0.9)	<0.001	2013–2017	−34.1 ( − 55.5, − 2.3)	0.038				−34.1 (−55.5, − 2.3)
Atlanta (Metropolitan)	1975–1991	3.4 (1.0,5.9)	0.006	1991–2014	−6.0 ( − 7.3, − 4.6)	<0.001	2014–2017	−38.8 ( − 65.0,7.0)	0.083	−31.8 (−54.5,2.1)

### Variations in suicide trends by sex

**Fig. 2:**
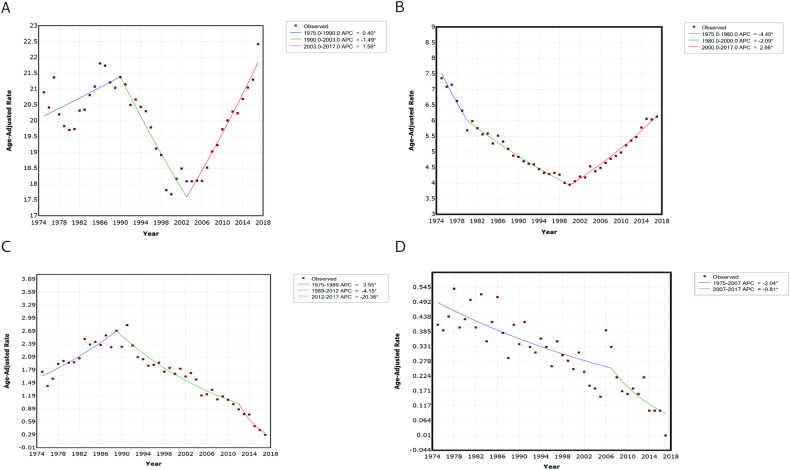
Age-adjusted suicide rate per 100,000 person-years and suicide trend varied by sex in the past 40 years. Trends of suicide among the total US population by male (**A**) and female (**B**). Trends of cancer-related suicide by male (**C**) and female (**D**). APC annual percentage change. *Indicates that the APC is significantly different from zero at the alpha = 0.05 level.

We further examined the profile and variations in suicide trends by sex between patients with cancer and the general US population (Fig. 2). In the general US population, we identified two joinpoints for males; suicide rates increased from 1975 to 1990, then considerably decreased from 1990 to 2003, but have been markedly increasing since then (Fig. 2A). Among females in the general US population, suicide rates decreased from 1975 to 2000, but have markedly increased in the past 17 years (Fig. 2B). In contrast, the cancer-related suicide trends showed significantly different patterns. Notably, cancer-related suicide rates among female patients have been consistently decreasing over the past 40 years, with a remarkable decrease observed since 2007. Overall, cancer-related suicide rates have decreased in both male and female patients in recent years, yet the suicide rate among the total US population is increasing. Moreover, in the most recent five years, male patients exhibited a greater decline in cancer-related suicide rates compared to female patients (Table 2).

### Variations in Suicide Trends by Race

**Fig. 3:**
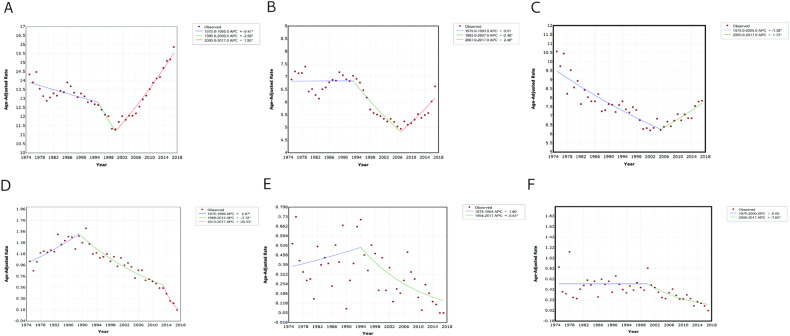
Age-adjusted suicide rate per 100,000 person-years and suicide trend varied by race in the past 40 years. Trends of suicide among the total US population by Caucasian (**A**), African American (**B**), and Other (American Indian/AK Native, Asian/Pacific Islander) (**C**). Trends of cancer-related suicide in the US by Caucasian (**D**), African American (**E**), and Other (American Indian/AK Native, Asian/Pacific Islander) (**F**). APC: annual percentage change. *Indicates that the APC is significantly different from zero at the alpha = 0.05 level.

We further analyzed the profile and variations in suicide trends by race between patients with cancer and the general US population (Fig. 3). In the general US population, the trends of suicide rates varied among different races. We identified two joinpoints for the Caucasian race; suicide rates gradually decreased from 1975 to 1995 and then significantly decreased from 1995 to 2000, followed by a marked increase since the year 2000 (Fig. 3A). In the African American race, we observed no significant changing trend in suicide rates from 1975 to 1993, followed by a decrease from 1993 to 2007, and a marked increase in the past decade (Fig. 3B, C). We found that the trend of cancer-related suicide rates among Caucasian patients was similar to the overall trend among all patients (Fig. 3D, E). Among African American patients, cancer-related suicide rates gradually increased from 1975 to 1994 and then markedly decreased since 1994. In summary, cancer-related suicide rates among Caucasian and African American have been decreasing in recent years, each follows a different pattern, contrasting with the increasing rates observed among the general US population. Specifically, from 2013 to 2017, Caucasian patients exhibited a larger decline in cancer-related suicide rates compared to other races (Table 2).

### Variations in Suicide Trends by Age

We compared the profile and variations of suicide trends by age between patients with cancer and the general US population (Fig. S1). In the general US population, suicide rates exhibited various patterns across different age groups. Among individuals aged 15 to 49 years, suicide rates gradually decreased from 1975 to 1996, then markedly decreased from 1996 to 2000, and subsequently increased significantly thereafter (Fig. S1A–E). In contrast, we observed significantly different patterns of cancer-related suicide trends compared to the general US population. Among patients aged 15 to 49 years, cancer-related suicide rates gradually decreased from 1998 to 2010, followed by a marked decrease thereafter (Fig. S1F). Similarly, among patients aged 50 to 59 years, cancer-related suicide rates gradually decreased from 1975 to 2002, followed by a marked decrease thereafter (Fig. S1G). Interestingly, a similar changing trend was observed in terms of cancer-related suicides among individuals aged 60 to 69 years and 70 to 79 years (Fig. S1H, I). We found that cancer-related markedly increased from 1975 to 1988 among these age groups, consistent with the trend observed in the overall US population. However, there has been a marked decrease in suicide rates since then (Fig. S1J). From 2013 to 2017, patients aged 60–79 years and 15–49 years exhibited a larger decline in cancer-related suicide rates compared to those in other age groups.

## Discussion

In our study, we performed in-depth analyses of the trends of cancer-related suicide in the US over a 40-year period. To the best of our knowledge, this is the first study to depict the changing patterns of cancer-related suicide trends and their variations based on the characteristics of patients and to compare them with the general US population. By examining the data by sex, race, age, and registry center, we observed variations in cancer-related suicide rates among different subgroups of patients. One significant finding is the decreasing trend of cancer-related suicide rates among patients since 1989, with a remarkable decrease in the most recent five years. While our study does not establish a causal relationship, it suggests that improvements in psychosocial care over the last few decades, along with advancements in cancer treatment and prevention, may have contributed to this downward trend. It is important to note that despite this decreasing cancer-related trend among patients, overall suicide rates in the general US population have increased.

The cancer-related suicide rate is estimated to be double that of the general population in the United States [19]. Notably, the risk of suicide in men is significantly higher compared to women [20]. This heightened cancer-related suicide risk remains elevated for up to 15 years following their diagnosis [7]. Previous studies have shown that multiple potential contributing factors increase the cancer-related suicide risk, including older age at diagnosis, lower educational level, previous attempted suicide, being unmarried, and psychological or psychiatric disorders [21–23]. Identifying certain key features early (hopelessness, low mood, feeling burdened by others, regret, loss of goals and meaning) may help in providing more focused care and in closely screening to assess associated risk factors [24–26]. The finding of an increasing trend of cancer-related suicide in the US followed by a peak in 1989 and subsequent descent is interesting. The significant decline in suicide rates, especially among male patients, Caucasian patients, and those aged 60 to 69 years in recent years adds to the complexity of the trend. We speculate that this is attributable to the following reasons. Firstly, there was a bottleneck in antitumor medicine for a long period since chemotherapy was first introduced in 1948 [27]. Secondly, as advancements were made in cancer treatment and surgical techniques, the landscape of cancer care began to change. Traditional open surgery can have several challenges and potential complications including intensive postoperative pain, significant trauma, slow healing, impacts on cardiopulmonary function, prolonged hospitalization, and high costs, which could seriously affect the quality of life of patients undergoing such procedures [28]. Thirdly, the financial burden associated with cancer care cannot be overlooked. The financial strain experienced by patients with cancer may extend beyond medical expenses to include caregiving, transportation, supplies, and childcare. Moreover, compared to individuals without a cancer history, those with cancer are more likely to face unique psychological and behavioral challenges, including emotional distress and material, medical, and financial adversity [29, 30].

The sharp slump in the rate of cancer-related suicide between 2013 and 2017 may be attributed to several factors. The expansion of Medicaid has been associated with increased access to healthcare services and improved affordability of medications and treatments for many individuals, including patients with cancer [31, 32]. In addition to the promising advances in medical treatments for malignancies, this period witnessed an evolving role of psycho-oncology care, palliative care, and hospice care, leading to the promotion and increased utilization of these services by patients with cancer, enhancing their overall quality of life [9–11, 33, 34]. Furthermore, the development of integrated care models, including collaborative care models, has provided a more comprehensive and coordinated approach to cancer care [35–38]. The implementation of all these measures may have contributed to the decline in cancer-related suicide rates between 2013 and 2017.

Since 2020, the COVID-19 pandemic has profoundly reshaped healthcare systems around the world, significantly impacting people’s health and wellbeing, economic development, and the routine functioning of society [39, 40]. Countries implemented various measures such as isolation, social distancing, and movement restrictions. Additionally, the overwhelming demand on healthcare services has led to delays in cancer patients receiving surgeries or chemotherapy treatments [41, 42]. This situation has markedly increased anxiety and depression among cancer patients, consequently elevating the suicide rate [43, 44]. This scenario underscores the unpredictable nature of changes in the cancer-related suicide risk trends. Sudden public health crises can lead to significant fluctuations in suicide rates. Therefore, it is imperative to establish an efficient cancer treatment system, preparing and responding effectively to any future public health emergencies of international concern.

Zaorsky et al. reported an overall suicide rate of 28.58 per 100,000 person-years among patients with cancer, significantly higher than the suicide rates reported in the general population [6]. They also found that the risk of suicide among patients with cancer was 4.4 times greater than that of the general population, which is broadly consistent with our previous work [45]. These findings highlight the urgent need for addressing the mental health issues of patients with cancer and to provide appropriate support and intervention strategies. Furthermore, the primary objective of our current study was to examine the trends in cancer-related suicides over recent decades using joinpoint models. We aimed to analyze the trends in cancer-related suicides over time rather than directly comparing the rates to those observed in the general population, or discussing the absolute numbers reported by Zaorsky et al. Nevertheless, our study aligns with the broader understanding that patients with cancer face a significantly higher risk of suicide and emphasizes the need for continued efforts to improve mental health support and care for these patients.

Several studies suggest a link between suicide risk and the type or severity of cancer. A Swedish cohort study conducted from 1965 to 1999 revealed that female patients with cancer had a higher suicide risk compared to male patients and that there was a strong inverse correlation between survival and suicide rate. This suggests that cancer types with a worse prognosis may be associated with higher suicide rates [46]. Choi et al. ’s study conducted in South Korea also indicated that suicide risk among patients with cancer varied according to the anatomical site of the cancer [47]. However, it did not find an association between cancer-related suicide risk and the prognosis of cancer. Anderson et al. reviewed data from the SEER database regarding patients diagnosed with cancer of the digestive system from 2000 to 2014, revealing that patients with pancreatic and esophageal cancer had more than five times the risk of suicide compared to the general population, while those with other digestive system cancers had about twice the risk of suicide compared to the general population [48]. This suggests that the specific type of cancer can affect suicide risk, with a worse prognosis potentially contributing to a higher risk, possibly due to feelings of depression and hopelessness accompanying the challenging prognosis [26, 49]. A study by Kahn et al. analyzed data from the Mental Health Research Network also reported that patients diagnosed with cancer with a poor prognosis in the past year had a nearly five-fold increased risk of suicide compared to the general population, while cancers with an average five-year survival rate of > 70% did not significantly increase the suicide risk [50]. These findings highlight the importance of tailoring support and prevention strategies based on the prognosis of the cancer. Patients with cancers that have a very poor prognosis, such as pancreatic cancer, should receive enhanced care, support, and psychological counseling.

Suicide and attempted suicide represent complex behaviors influenced by numerous proximal and distal risk factors [51]. These factors can be organized within explanatory models, potentially aiding in the comprehension of suicidal individuals and enhancing the evaluation of suicide risk [52]. Kees et al. seminally analyzed the stress diathesis model of suicide and investigated the neurobiological basis of suicide in depth [53]. Suicide was shown to result from interactions between state-dependent (environmental) stressors and trait diathesis or susceptibility to suicidal behavior. Kees van Heeringen’s model emphasizes the role of the brain’s stress-response system in suicide risk, particularly focusing on how neurobiological changes may predispose individuals to suicidal behavior. This model could be particularly relevant in explaining the psychological distress and heightened cancer-related suicide risk, as it integrates biological, psychological, and social factors [54]. For example, early adversity may influence suicide risk by shaping neural circuits. Dysregulation of stress response and the potential cytotoxic effects of excessive concentrations of corticotropin-releasing hormone and glucocorticoids may play a role [55]. Therefore, alongside cognitive and emotional strategies, incorporating neurobiological assessments and interventions is crucial in the prevention of suicidal tendencies. Neuroimaging can delineate brain regions and networks associated with suicide risk and can be a way to track the effects of interventions targeting such specific brain regions and neural networks. Combined with genomic marker technology, it helps guide personalized interventions to prevent suicidal behavior. The theory of computational psychiatry is starting to venture into bold forecasts regarding real-world dynamics. To advance the next wave of computational psychiatry, it’s essential to integrate modeling and measurement techniques from related disciplines, such as network and complex systems methodologies and digital phenotyping [56, 57].

By providing insights into the temporal trends of cancer-related suicides, our study contributes to the existing knowledge on the subject and may inform future interventions and support strategies for patients with cancer at risk of suicide. Our study has several strengths. First, it is the largest and most comprehensive in characterizing the profile of cancer-related suicide trends over a 40-year period. By studying a diverse population of patients with cancer, rather than focusing on specific complications or single-system malignancies, we enhance the generalizability and applicability of our findings. Second, this study is the first to describe the 40-year changing pattern of cancer-related suicide rates through joinpoint regression analysis. These joinpoints allow us to accurately capture and describe the changing patterns over time. This approach provides a comprehensive map of the cancer-related suicide rate patterns among patients and, by comparing these patterns with the suicide rates in the general US population, further enhances our understanding of the unique challenges faced by these patients.

### Limitations

Our study has several limitations that should be acknowledged. We have extensively reviewed a large amount of data and literature related to the SEER program [58, 59]. However, the findings of our study indicate a potential bias. On the one hand, SEER pathology terminology has undergone changes over time due to advances in tumor classification, which has led to long-term challenges regarding the comparability of SEER data captured over more than 40 years. Due to advances in diagnostic testing, recent pathologic diagnoses captured by the SEER database are expected to be more precise than those made 40 years ago. This situation poses a challenge in terms of aligning with new diagnostic criteria and tumor classification systems. Moreover, the National Cancer Institute (NCI) adopted the new behavioral code ICD-O-3 in 2021, which may impact our study. On the other hand, source data inaccuracies can arise from data coding errors transmitted to the NCI by regional registration authorities or incorrect data provided to regional registration services for coding. In addition, although SEER accounts for the proportion of each ethnic group and even oversamples certain racial and ethnic minorities when sampling populations to improve the representation of diverse populations, the proportion of the US population has undergone significant changes over the past 40 years due to various social factors such as economic development and increased migration. This demographic shift presents another limitation to our study. According to the reviewers’ comments, our results should be considered in the context of the influence of the prevalence of cancer over the period. Despite the stable or slightly increasing prevalence of cancer from 1990 to 2017 [60], our study observed a decreasing trend in cancer-related suicide rates. This finding is particularly significant as it suggests that the decrease in suicide risk is not a result of a reduced number of individuals living with cancer and might have been underestimated in our study. Besides, any underestimation of suicide rates in our study is likely to be similar in patients with cancer and the geeral population, which reduces the likelihood of major deviations when calculating relevant data. The observed association between cancer and suicide may be confounded by psychiatric disorders and medication use, factors we were unable to control for in the current work.

### Supplementary information

Legend of Figure S1

Figure S1

## Data Availability

All supporting data are included in the manuscript and supplemental files. Additional data are available upon reasonable request to the corresponding author.
