# Supplementary material for: Trends in cancer-related suicide in the United States: a population-based epidemiology study spanning 40 years of data
Source: Transl Psychiatry. 2024 May 27;14:213. doi: 10.1038/s41398-024-02917-9 (PMC11130301; doi:10.1038/s41398-024-02917-9)
Supplement: Supplementary file 1 — Legend of Figure S1 [file 41398_2024_2917_MOESM1_ESM.docx]

**Figure S1. Age-adjusted suicide rate per 100,000 person-years and suicide trend varied by age in the past 40 years**. Trends of suicide among the total US population by 15–49 years (A), 50–59 years (B), 60–69 years (C), 70–79 years (D), 80+ years (E); Trends of cancer-related suicide in the US by 15–49 years (F), 50–59 years (G), 60–69 years (H), 70–79 years (I), 80+ years (J). APC: annual percentage change. *Indicates that the APC is significantly different from zero at the alpha = 0.05 level.
