# Supplementary figures and images for: Trends in cancer-related suicide in the United States: a population-based epidemiology study spanning 40 years of data
Source: Transl Psychiatry. 2024 May 27;14:213. doi: 10.1038/s41398-024-02917-9 (PMC11130301; doi:10.1038/s41398-024-02917-9)

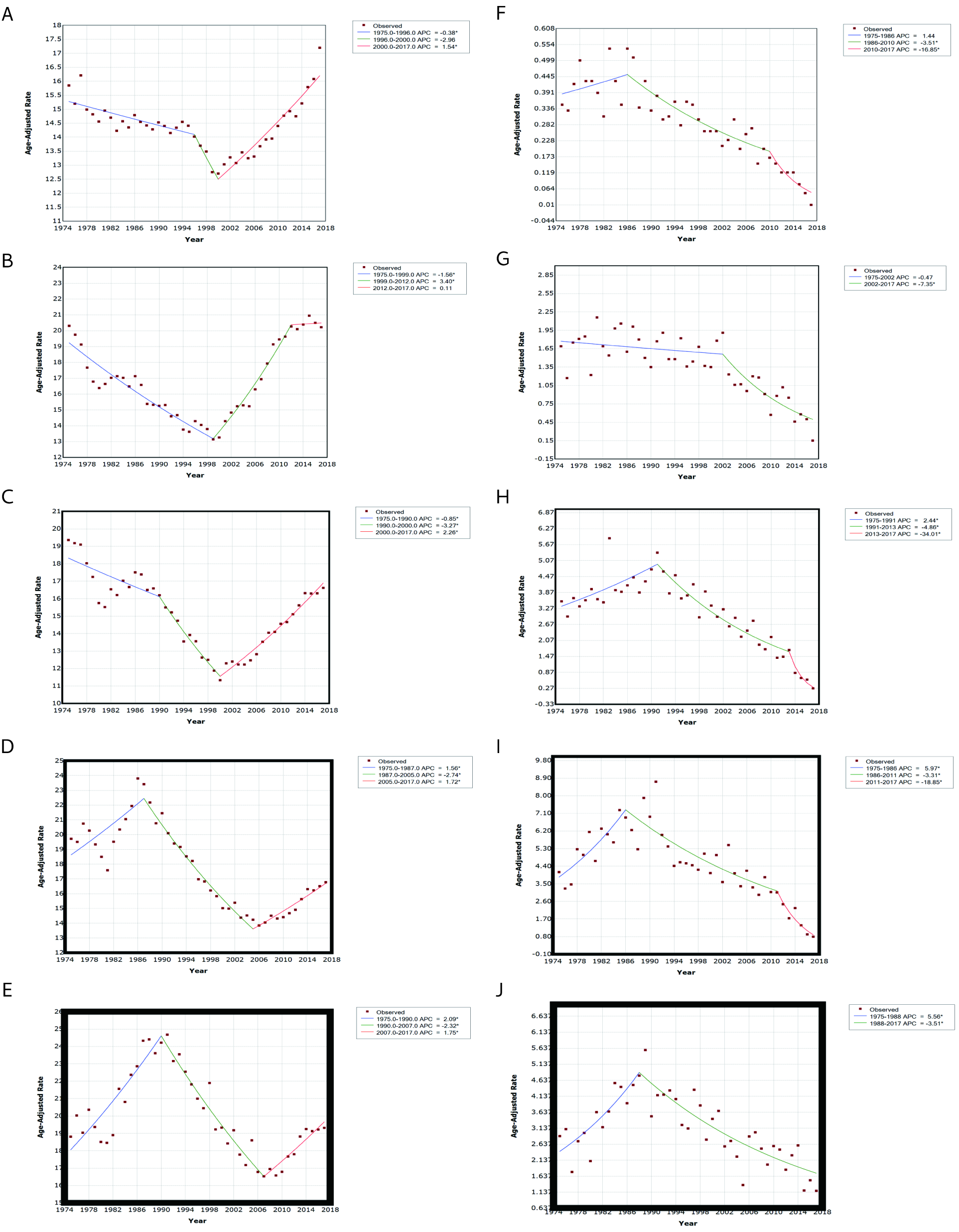

Supplement: Supplementary file 2 — Figure S1 [file 41398_2024_2917_MOESM2_ESM.tif]
